# Supplementary material for: Sensory Neurons Release Cardioprotective Factors in an In Vitro Ischemia Model
Source: Biomedicines. 2024 Aug 15;12(8):1856. doi: 10.3390/biomedicines12081856 (PMC11351881; doi:10.3390/biomedicines12081856)
Supplement: Supplementary file 1 [file biomedicines-12-01856-s001.zip › biomedicines-3136534-supplementary.pdf]

Sensory Neurons Release Cardioprotective Factors in an In Vitro Ischemia Model – Supplemental material

Clara Hoebart [1], Attila Kiss [2], Bruno K. Podesser [2], Ammar Tahir [3], Michael J. M. Fischer [1,*,†] and Stefan Heber [1,†]

[1] Institute of Physiology, Center for Physiology and Pharmacology, Medical University of Vienna, 1090 Vienna, Austria; clara.hoebart@meduniwien.ac.at (C.H.); stefan.heber@meduniwien.ac.at (S.H.)

[2] Center for Biomedical Research and Translational Surgery, Medical University of Vienna, 1090 Vienna, Austria; attila.kiss@meduniwien.ac.at (A.K.); bruno.podesser@meduniwien.ac.at (B.K.P.)

[3] Division of Pharmacognosy, University of Vienna, 1090 Vienna, Austria; ammar.tahir@univie.ac.at

* Correspondence: michael.jm.fischer@meduniwien.ac.at; Tel.: +43-1-40160-31411

† These authors contributed equally to this work.

Supplementary figures

DRG neuron's protection of cardiomyocytes against ischemia-reperfusion is not mediated by calcitonin gene-related peptide nor influenced by botulinum toxin A

To probe whether calcitonin gene-related peptide release might explain an increased ischemia-reperfusion tolerance of cardiomyocytes, single- and co-cultures were treated with the calcitonin gene-related peptide receptor antagonist olcegepant. There was no evidence that treatment with olcegepant influenced the effect of DRG neurons on cardiomyocyte survival differently depending on whether assessed under ischemic or control conditions (three-way interaction $p = 0.32$). In ischemia-reperfusion the application of olcegepant did not change the influence sensory neurons had on the survival of cardiomyocytes ($p = 0.30$). Additionally, there was no main effect of olcegepant treatment in ischemia-reperfusion conditions (main effect $p = 0.16$, Suppl. Fig. S1 A,B).

Whether exocytosis from sensory neurons mediates their protective effect was probed by the neurotoxin botulinum toxin A. There was no evidence that treatment with botulinum toxin A influenced the effect of sensory neurons on cardiomyocyte survival differently depending on whether assessed under ischemia-reperfusion or control conditions (Three-way interaction $p = 0.51$). In case sensory neurons release protective molecules via exocytosis, one would expect the protective effect of sensory neurons to be diminished in the presence of botulinum toxin A. However, there was even a trend towards higher increase of cardiomyocyte survival due to sensory neurons in the presence of botulinum toxin A in ischemia-reperfusion ($p = 0.064$). Irrespective of whether sensory neurons were present or not, botulinum toxin A increased the survival probability of cardiomyocytes in the context of ischemia-reperfusion (main effect $p < 0.001$, Suppl. Fig.S1 C,D).

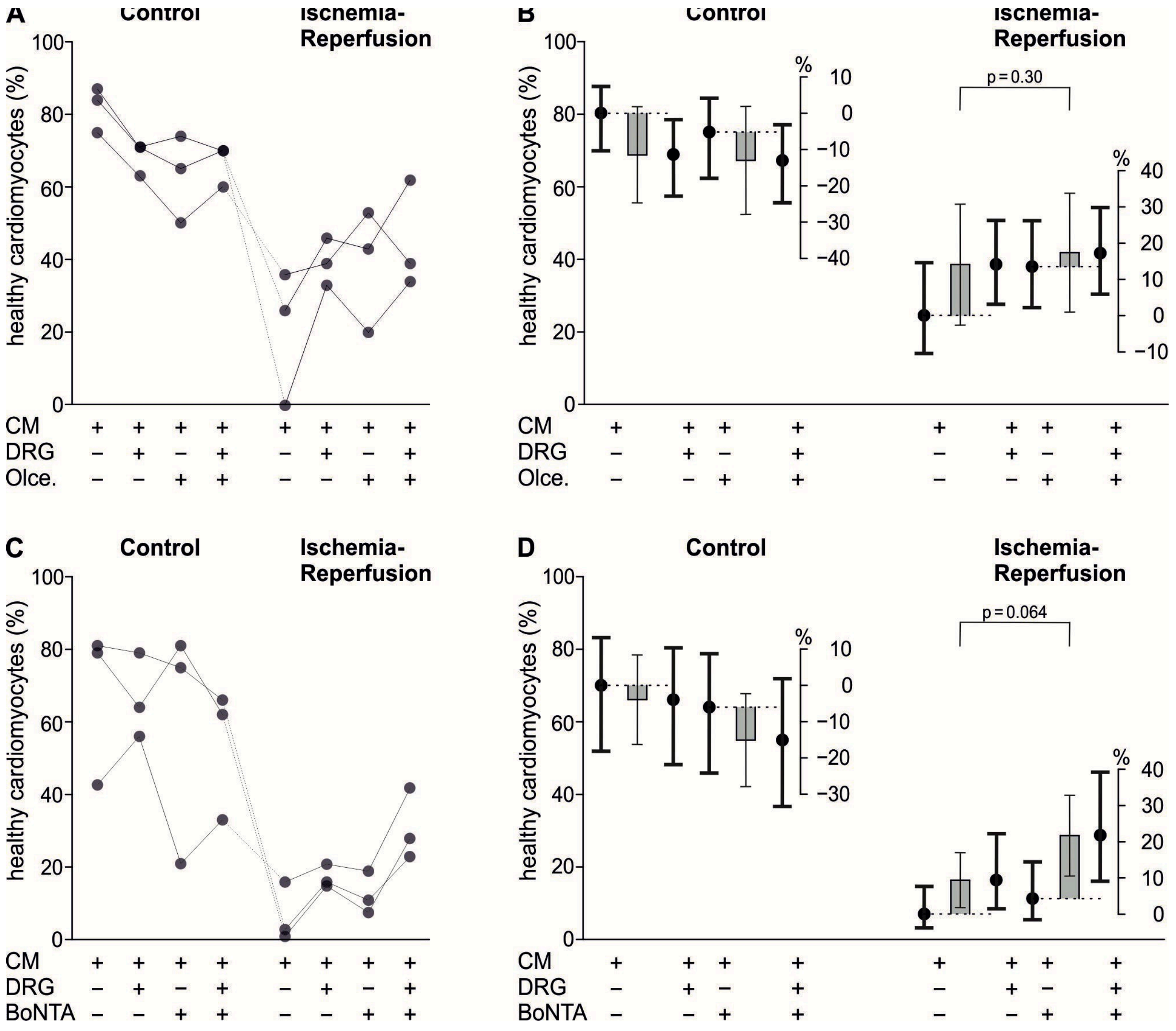

Supplementary Figure S1: Calcitonin gene-related peptide receptors and vesicular exocytosis do not mediate increased ischemia tolerance of cardiomyocytes. A: Cardiomyocytes (CM) and cardiomyocytes + DRG neurons (DRG) were treated with the calcitonin gene-related peptide receptor antagonist olcegepant (Olce.) or without. Percentage of healthy cardiomyocytes at the end of the cellular model for ischemia-reperfusion. Every dot represents the mean of one experiment and experiments conducted in parallel are connected. In every of the 3 independent experiments, the cardiomyocytes from one mouse were distributed amongst the experimental conditions. For co-culture they were added on top of the DRG from another mouse per experiment. **B:** Estimated survival probabilities for each group are shown as black dots ± 95% CI and estimated differences are shown as gray bars ± 95% CI. **C:** Cardiomyocytes and cardiomyocytes + DRG neurons were treated with botulinum toxin A (BoNTA) or without. Percentage of healthy cardiomyocytes at the end of the cellular model for ischemia-reperfusion. Every dot represents the mean of one experiment and experiments conducted in parallel are connected. In every of the 3 independent experiments, the cells from one mouse were distributed amongst the experimental conditions. For co-culture they were added on top of the DRG from another mouse per experiment. **D:** Estimated survival probabilities for each group are shown as black dots ± 95% CI and estimated differences are shown as gray bars ± 95% CI.

Cytokine production was influenced by the presence of sensory neurons

Next, it was tested whether cytokines are altered by ischemia or the presence of sensory neurons, as these are common soluble factors of cell-cell communication. Therefore, 106 cytokines were measured in the supernatants of single- and co-cultures at the end of the cellular ischemia-reperfusion model and in control conditions. The fold changes of cytokines in ischemia-reperfusion vs. control conditions displayed for single- and co-culture show that some cytokines were more abundant after ischemia-reperfusion, and even more upregulated in co-culture (Suppl. Fig. S2a). The correlations of the groups with each other show that there are more cytokines that are differentially abundant (red dots) with the addition of sensory neurons than with ischemia (Suppl. Fig. S2b). Two cytokines stood out from the analysis, by being different in ischemia and that the presence of sensory neurons increased this difference. This applies to osteopontin, which was further increased in the presence of sensory neurons, and APCS (serum amyloid P component) which was further decreased in the presence of sensory neurons (Suppl. Fig. S2c). The cytokines were also subjected to a pathway analysis using the ExpressAnaylst tool[1], which found the Janus kinase - signal transducer and activator of transcription (Jak-STAT) pathway as the most important hit. Fold changes of cytokines associated with the Jak-STAT pathway and their density distribution show that cytokines associated with the Jak-STAT pathway were decreased in abundance by the addition of sensory neurons in control conditions and increased in abundance by ischemia-reperfusion conditions in cardiomyocyte cultures. However, the pathway regulation in presence of sensory neurons in ischemic conditions largely matched the sensory neurons (Fig. S5d).

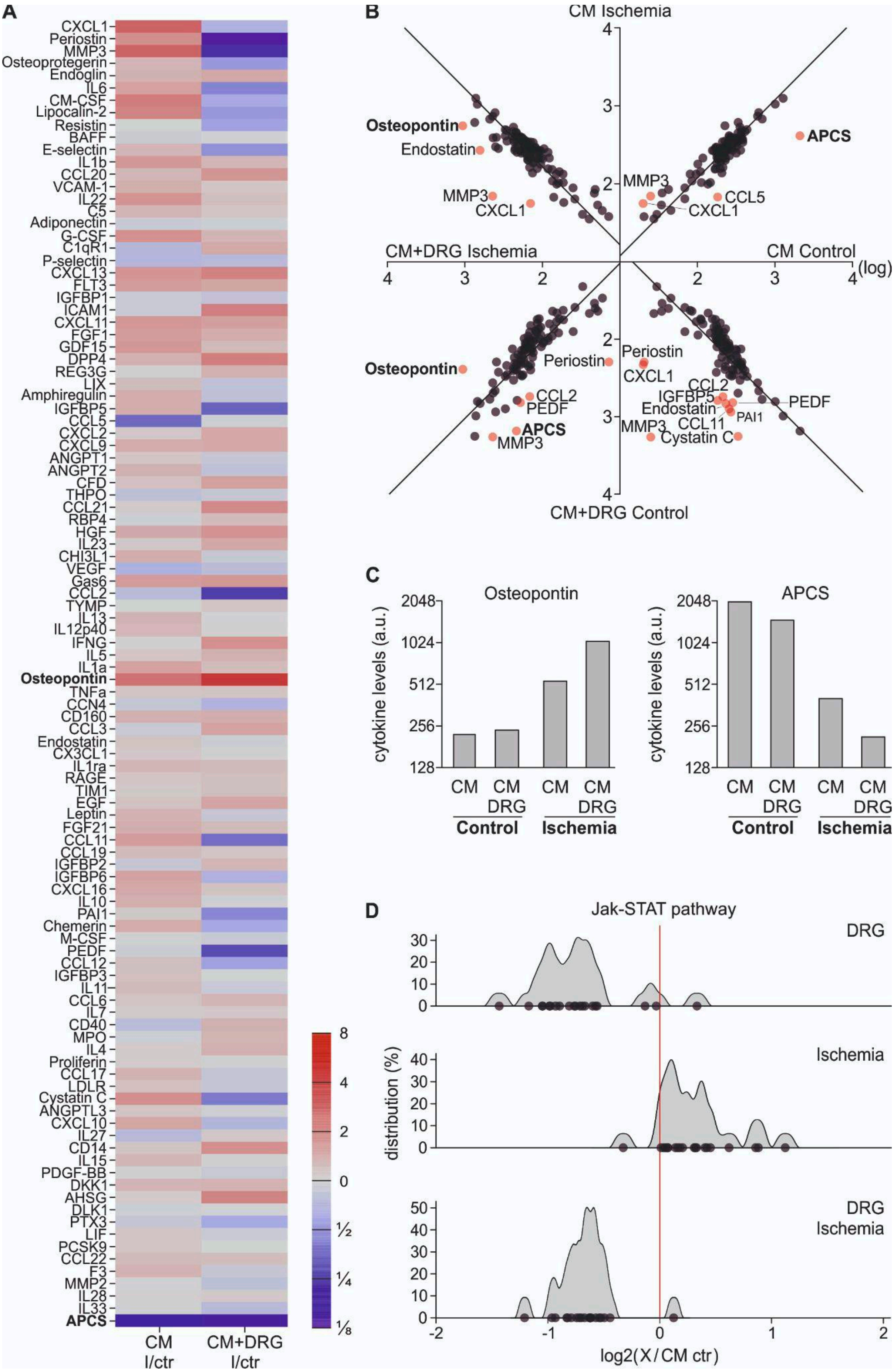
A
CXCL1
Periostin
MMP3
Osteoprotegerin
Endoglin
IL6
CM-CSF
Lipocalin-2
Resistin
BAFF
E-selectin
IL1b
CCL20
VCAM-1
IL22
C5
Adiponectin
G-CSF
C1qR1
P-selectin
CXCL13
FLT3
IGFBP1
ICAM1
CXCL11
FGF1
GDF15
DPP4
REG3G
LIX
Amphiregulin
IGFBP5
CCL5
CXCL2
CXCL9
ANGPT1
ANGPT2
CFD
THPO
CCL21
RBP4
HGF
IL23
CHI3L1
VEGF
Gas6
CCL2
TYMP
IL13
IL12p40
IFNG
IL5
IL1a
Osteopontin
TNFa
CCN4
CD160
CCL3
Endostatin
CX3CL1
IL1ra
RAGE
TIM1
EGF
Leptin
FGF21
CCL11
CCL19
IGFBP2
IGFBP6
CXCL16
IL10
PAI1
Chemerin
M-CSF
PEDF
CCL12
IGFBP3
IL11
CCL6
IL7
CD40
MPO
IL4
Proliferin
CCL17
LDLR
Cystatin C
ANGPTL3
CXCL10
IL27
CD14
IL15
PDGF-BB
DKK1
AHSG
DLK1
PTX3
LIF
PCSK9
CCL22
F3
MMP2
IL28
IL33
APCS
CM I/ctr
CM+DRG I/ctr
8
4
2
0
½
¼
⅛
B
CM Ischemia
CM+DRG Ischemia
CM Control
CM+DRG Control
(log)
Osteopontin
Endostatin
MMP3
CXCL1
APCS
CCL5
Periostin
CCL2
PEDF
IGFBP5
Endostatin
CCL11
PAI1
Cystatin C
C
Osteopontin
APCS
cytokine levels (a.u.)
2048
1024
512
256
128
CM
CM DRG
Control
Ischemia
D
Jak-STAT pathway
DRG
Ischemia
DRG Ischemia
distribution (%)
log2(X/CM ctr)

Supplementary Figure S2: Cytokines released in the ischemia-reperfusion model. A: Heatmap of cytokines sorted by their abundance from lowest (top) to highest (bottom). The first column shows the fold change of the amount of cytokine released by cardiomyocytes in ischemia-reperfusion conditions compared to control conditions. The second column shows the same for the co-culture of sensory neurons with cardiomyocytes. Red indicates an increase and blue a decrease. Changes are log2-scaled. **B:** Every quadrant provides the linear correlation of two groups on a log scale and cytokines with relevant differences between the two axes are shown in red. **C:** Bar chart of cytokine levels of osteopontin and APCS on a log2 scale in arbitrary units demonstrate a change by the ischemia-reperfusion which is larger in the presence of sensory neurons. **D:** Ridgeline plot of the fold change to the cardiomyocyte control group of cytokines found to be involved in the Jak-STAT pathway, on a log2 scale. The red line indicates no change, gray dots show fold change to cardiomyocytes control of single cytokines and the curve shows the density plot of these values. The analysis was based on the pooled solutions generated by n = 6 animals in total.

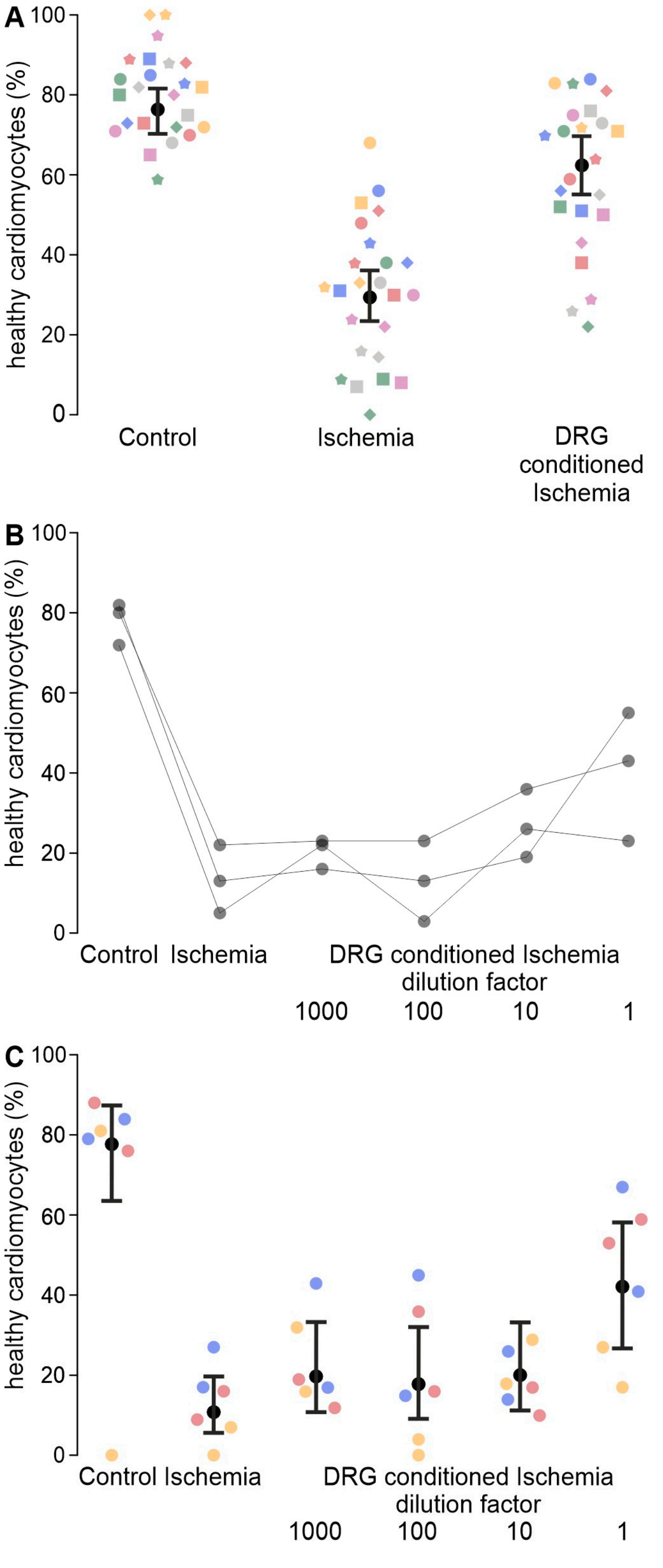

Supplementary Figure S3: Experimental variation of cardioprotective effect of DRG-conditioned ischemic solution. A) Cardiomyocytes were treated with DRG-conditioned ischemic solution. Estimated survival probabilities for each condition are shown as black dots, with each unique combination of a shape and a color representing one experiment. **B)**

Cardiomyocytes were treated with DRG-conditioned ischemic solution in different dilutions by a factor of 1000, 100 and 10. Estimated survival probabilities for each condition are shown as black dots with experimental days being connected by a line. **C)** Estimated survival probabilities are shown as black dots ± 95% CI, dots indicate single wells of experimental days, each color represents data from a specific animal.

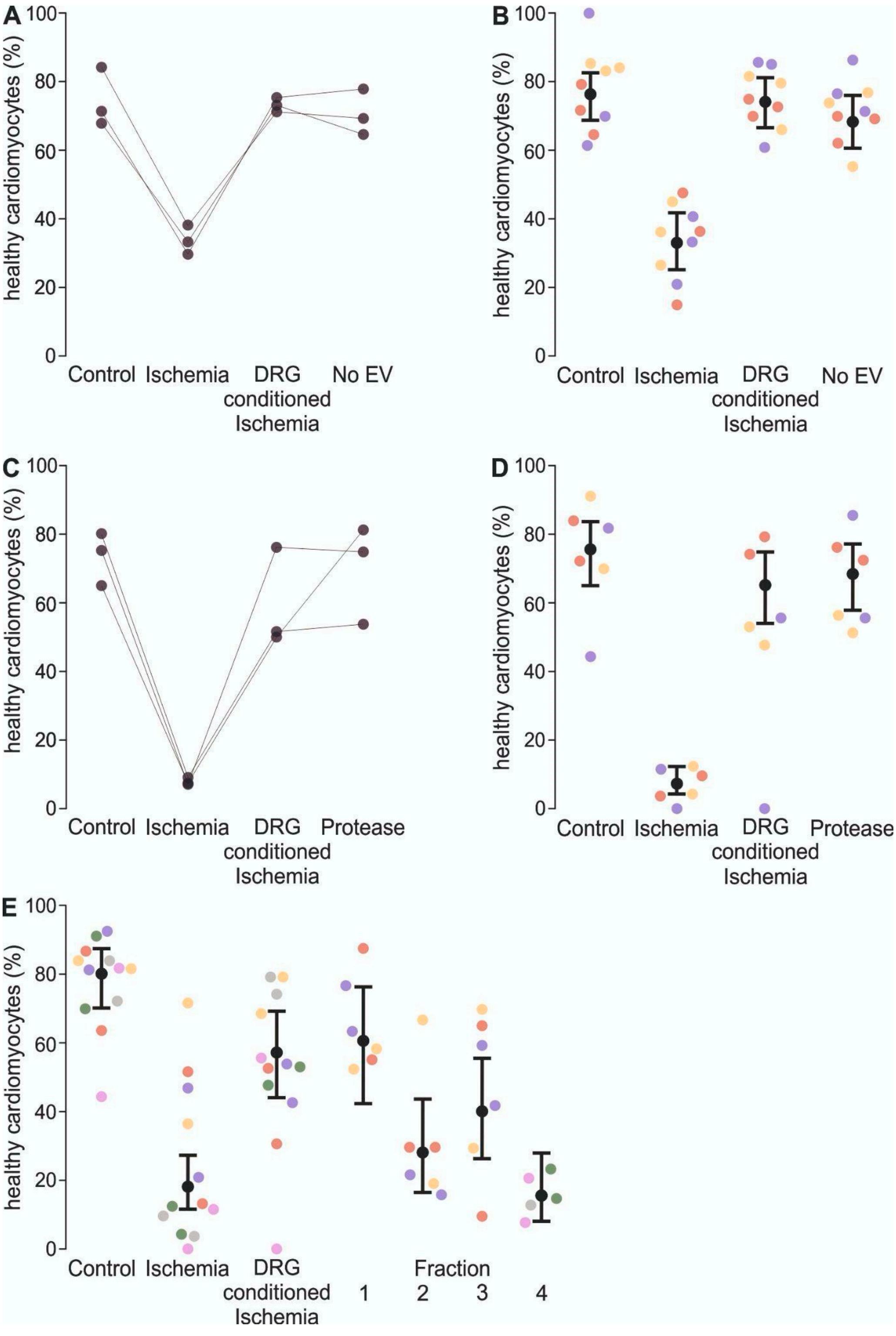

Supplementary Figure S4: Experimental variation of cardioprotective effect of various factors tested. A) Cardiomyocytes were treated with DRG-conditioned ischemic solution with and without extracellular vesicles. In panel A, symbols connected by a line represent results from one experimental day. **B)** Results from panel A, with each well represented by a symbol, symbols with identical color represent data from the same experimental day, i.e. from the same animal. **C)** Cardiomyocytes were treated with DRG-conditioned ischemic solution treated with protease or without. Symbols connected by a line represent results from

one experimental day. **D)** Results from panel C, with each well represented by a symbol, symbols with identical color represent data from the same experimental day, i.e. from the same animal. **E)** Cardiomyocytes were treated with DRG-conditioned ischemic solution or with 4 different fractions of the DRG-conditioned ischemic solution, which were obtained using a C18 column and different eluents. For fraction 1 it was water and for the other fractions increasing concentrations of acetonitrile, namely 30%, 60% and 100% for fractions 2-4, respectively. In panels B, D and F, estimated survival probabilities are shown as black dots ± 95% CI, colored dots indicate single wells of experimental days.

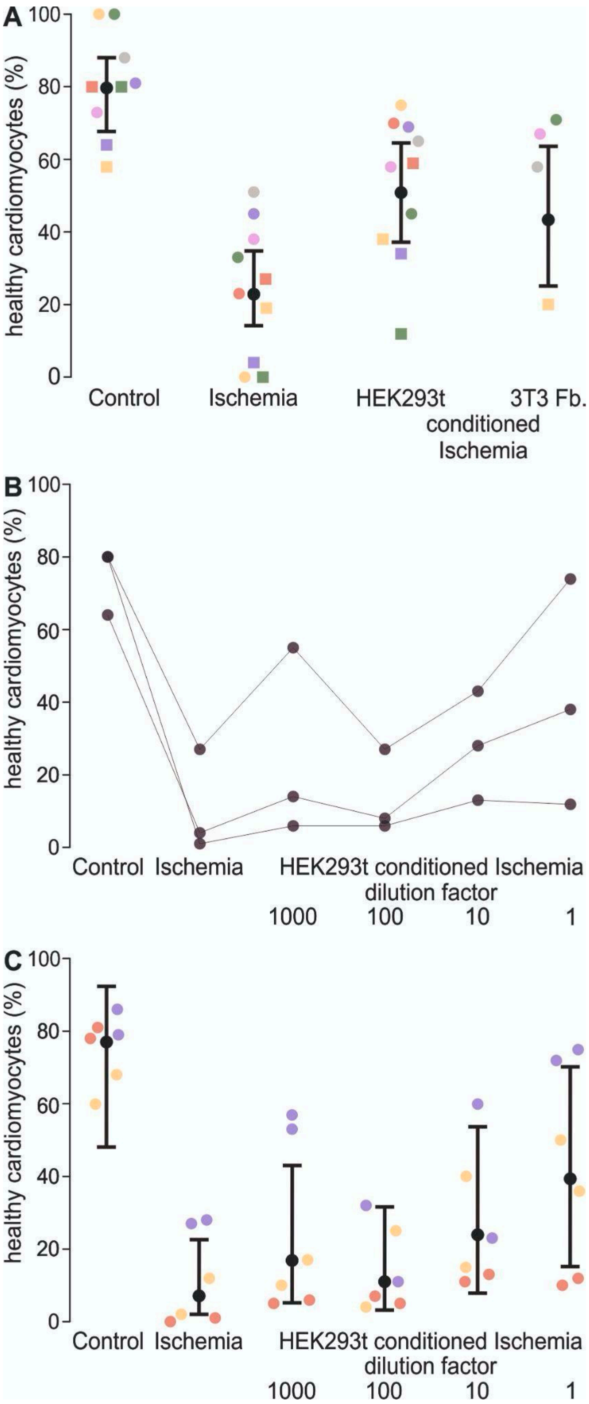

Supplementary Figure S5: Experimental variation of cardioprotective effect of cell lines and HEK293t-conditioned ischemic solution dilutions. A) The cell lines HEK293t and 3T3 fibroblasts conditioned the ischemic solution before it was applied to cardiomyocytes. Estimated survival probabilities for each cell line are shown as black dots ± 95% CI. Every different color-shape combination represents an independent experiment and also the number of animals used. **B)** The effect of the HEK293t-conditioned ischemic solution is dose- dependent, with dilutions by a factor of 1000, 100 and 10 shown. Estimated survival probabilities for each condition are shown as black dots with experimental days being connected by a line. **D)** Estimated survival probabilities are shown as black dots ± 95% CI, colored dots indicate single wells of experimental days, each day is represented by a color, which also represents number of animals used.

Supplementary tables

Metabolite	log2(FC)	-log10(p)	Metabolite	log2(FC)	-log10(p)
Unknown-168	-3.4	6.6	Dioctyl Phthalate	3.5	0.9
Unknown-47	4.5	6.4	Oleoyl-L-Carnitine	-3.0	0.9
Unknown-74	3.4	6.3	Unknown-204	1.5	0.9
Unknown-43	-4.0	6.1	Unknown-163	-3.1	0.9
Unknown-91	3.4	6.0	Unknown-55	-3.3	0.9
Unknown-115	3.9	5.9	Unknown-103	3.3	0.9
Unknown-210	3.5	5.6	Unknown-160	1.6	0.9
Unknown-131	3.5	5.4	Unknown-44	3.5	0.9
Unknown-196	3.5	5.3	PE 38:2	1.0	0.9
Unknown-155	-4.1	5.0	PE O-41:2	1.1	0.9
Unknown-16	-3.7	5.0	Unknown-110	3.6	0.9
Unknown-232	3.6	4.9	PC O-40:2	1.2	0.9
Ganglioside GM3	-1.9	4.8	Acetyl tributyl citrate	1.3	0.9
Spermidine	4.9	4.6	Unknown-38	1.3	0.9
Nicotinamide	-3.9	4.4	Unknown-33	1.2	0.9
Adenosine	4.0	4.3	PE 34:0	1.1	0.9
MG 15:4	1.6	3.7	NAE 22:5	-1.4	0.9
inosine	4.0	3.6	Myristamidopropyl betaine	1.2	0.9
Hypoxanthine	-3.2	3.6	SM 41:2;O2	2.5	0.8
PC O-40:11	2.2	3.4	Unknown-133	1.2	0.8
PS 36:2|PS 18:1_18:1	2.4	2.8	PC O-35:2	2.2	0.8
PS 40:1	4.7	2.7	Unknown-112	1.2	0.8
NAE 24:5	1.0	2.6	Unknown-195	1.3	0.8

NAE 28:7	-6.5	2.6	DG 50:10	-1.2	0.8
DG 33:0	2.5	2.5	Unknown-72	-1.3	0.8
DG 16:0	2.4	2.5	DG 36:5	-1.3	0.8
PE O-35:2	2.4	2.3	PC O-41:2|PC O-23:1_18:1	2.2	0.7
PS 40:4	1.1	2.1	NAE 28:0	-1.3	0.7
CAR 18:1	1.1	2.0	SM 40:2;O2	1.2	0.7
NAE 18:4	-1.1	2.0	NAE 26:0	-1.3	0.7
PC O-38:7	1.9	2.0	Unknown-221	1.0	0.7
PC 42:2	2.7	1.9	Unknown-11	-1.1	0.7
PE P-36:2|PE P-18:1_18:1	1.7	1.9	Cer 36:1;O2|Cer 18:1;O2/18:0	-1.9	0.7
Unknown-123	-3.7	1.8	Unknown-101	1.2	0.7
PE P-34:2|PE P-18:1_16:1	2.0	1.8	LPE 18:1	1.1	0.7
PC 42:1	4.6	1.7	DG 32:0	1.3	0.6
PE P-36:3|PE P-18:1_18:2	1.3	1.7	PC O-39:1|PC O-21:0_18:1	1.0	0.6
DG 33:1	1.9	1.6	Unknown-152	-1.4	0.6
LPE O-18:2	1.4	1.6	DG 27:3	-1.1	0.6
DG 35:8	-6.7	1.6	ST 27:2;O	-1.1	0.5
PC O-43:1	2.7	1.6	Unknown-178	2.8	0.5
PS 34:1	1.4	1.5	TG 47:0|TG 15:0_16:0_16:0	-1.0	0.5
PE P-40:4|PE P-18:0_22:4	1.3	1.5	PC 40:2	1.1	0.5
TG 42:0;O|TG 16:0_16:0_10:0;O	3.7	1.4	DG 26:4	-1.0	0.5
PE P-34:1|PE P-16:0_18:1	1.2	1.4	Unknown-213	-2.3	0.5
LPC 16:0	2.3	1.4	Unknown-73	2.0	0.4

Unknown-98	2.5	1.4	Unknown-199	5.7	0.4
Unknown-87	-2.3	1.3	Unknown-12	-4.7	0.4
Unknown-79	2.2	1.3	Denatonium	3.0	0.4
Cer 33:2;O3|Cer 18:1;O2/15:1;O	-1.3	1.3	Gomisin H	2.4	0.4
PC O-41:1|PC O-23:0_18:1	2.9	1.3	Unknown-167	-2.1	0.4
Palmitoylcarnitine	2.2	1.3	Unknown-166	2.0	0.4
Unknown-170	-2.7	1.3	Diphenylphosphate	-2.0	0.4
LPC 18:1	1.3	1.2	Unknown-156	2.0	0.4
Unknown-21	2.0	1.2	Liquiritigenin	-2.0	0.4
Cer 34:1;O2|Cer 18:1; O2/16:0	-1.1	1.2	Unknown-122	-2.0	0.4
PE P-38:5|PE P-18:1_20:4	1.1	1.2	Unknown-119	-5.4	0.4
Unknown-227	2.1	1.2	Unknown-159	-5.4	0.4
Unknown-231	2.2	1.2	Unknown-205	5.0	0.4
Unknown-121	1.8	1.1	Unknown-108	-4.8	0.4
DG 36:3|DG 18:1_18:2	1.3	1.1	Unknown-90	-4.0	0.4
Cer 36:2;O2|Cer 18:2; O2/18:0	-2.3	1.1	Unknown-176	-3.8	0.4
Unknown-75	1.7	1.1	Unknown-139	-3.7	0.4
DG 40:10	2.4	1.1	Unknown-64	3.1	0.4
Unknown-229	2.0	1.1	DG 45:7	3.0	0.4
Unknown-49	1.7	1.1	Unknown-189	-2.9	0.4
LPE O-18:1	1.6	1.1	Unknown-31	-2.9	0.4
Unknown-30	-1.7	1.1	Unknown-116	-2.7	0.4
Unknown-94	-1.6	1.1	Unknown-177	2.7	0.4

PC O-34:1|PC O-18:1_16:0	1.2	1.0	Unknown-36	-2.6	0.4
Unknown-180	-1.7	1.0	Unknown-109	2.6	0.4
DG 19:0	2.5	1.0	Tri(butoxyethyl)phosphate	2.5	0.4
Unknown-175	1.8	1.0	Unknown-222	-2.5	0.4
Unknown-202	1.8	1.0	Unknown-165	-2.4	0.4
Unknown-219	1.8	1.0	8-acetamido-2-methyl-7-oxononanoic acid	2.3	0.4
SM 34:2;O2	1.3	1.0	Mono-iso-butyl phthalate	2.2	0.4
glycycoumarin	1.6	1.0	Unknown-3	2.1	0.4
HexCer 41:3;O3	1.2	1.0	Hemslecin A	-2.0	0.4
Atractylon	1.6	1.0	Unknown-174	2.0	0.4
Unknown-215	1.7	1.0	N-Methyl-2-pyrrolidone	-2.0	0.4
Unknown-150	-1.7	1.0	(S)-3-(4-hydroxyphenyl) chroman-7-ol	-2.0	0.4
Unknown-161	1.5	1.0	PC(16:0/18:1(9Z))	2.0	0.4
LPE 20:1	1.2	1.0	Unknown-42	2.0	0.4
PC O-39:5	2.1	0.9	Unknown-164	-2.0	0.4
methyl 3-(6-((4-formylpiperazin-1-yl)methyl)-3-hydroxy-4-oxo-4H-pyran-2-yl)-3-(4-((1-methyl-1H-imidazol-2-yl)methoxy) phenyl) propanoate	1.4	0.9	Unknown-194	-1.8	0.4
Unknown-218	1.4	0.9	Unknown-192	-1.7	0.4
Unknown-8	1.4	0.9	Unknown-102	-1.9	0.4
Unknown-212	-1.6	0.9	Unknown-52	-1.7	0.4
Gentiannine	-2.8	0.9	PS 38:4	1.2	0.4
Unknown-56	4.0	0.9	Unknown-105	-1.4	0.4
Unknown-173	2.8	0.9	Unknown-51	-1.3	0.4

Unknown-60	4.0	0.9	Unknown-234	1.4	0.4
Phthalic anhydride	-3.0	0.9	Unknown-203	1.5	0.3
Unknown-84	-3.7	0.9	Unknown-68	-1.1	0.3
Unknown-162	-4.4	0.9	2-acetoxy-4-pentadecylbenzoic acid	1.0	0.3
Unknown-37	-3.8	0.9	DG 47:7	-1.3	0.3
Unknown-151	4.1	0.9	Unknown-61	1.1	0.3
Triphenylphosphate	3.6	0.9	Unknown-86	-1.1	0.3
Unknown-32	-8.0	0.9	Unknown-179	-1.1	0.3
Unknown-97	-4.2	0.9	Unknown-198	-1.1	0.3
Unknown-138	-4.0	0.9	Unknown-92	-1.2	0.3
Unknown-23	3.1	0.9	Unknown-126	1.1	0.3
Unknown-144	-3.2	0.9	Unknown-211	-1.0	0.3
Unknown-124	2.9	0.9	TG 34:0|TG 8:0_8:0_18:0	1.1	0.2
Icaritin	-2.9	0.9	Unknown-18	-2.6	0.2
Leucine	-2.9	0.9	DG 48:7	2.2	0.2
Unknown-80	-3.1	0.9	DG 23:4	-1.0	0.2
Unknown-128	-3.7	0.9	Unknown-125	-2.5	0.1
Unknown-187	1.6	0.9	Solasodin	1.4	0.1
Unknown-59	-4.3	0.9	Unknown-2	-1.1	0.1
Dioctyl Phthalate	3.5	0.9	LPC 18:1; Plasma ID-2760	1.1	0.1

Supplementary Table S1: Detected features in DRG-conditioned ischemic and external solution. Metabolites and lipids were matched to reference spectral libraries, features that could not be identified nor annotated are labeled as unknown. A total of 681 metabolites and lipids were annotated or identified, of these the 222 metabolites with an at least twofold different value are listed. The table indicates the log2 of the fold change (FC), calculated by dividing the measurement from DRG-conditioned ischemic solution by the measurement from DRG-conditioned external solution. Further, the negative log10 of the p-value of this observation is reported. The list is sorted by p-value, the upper 49 identified metabolites and lipids of this list are significantly regulated (–log10(0.05)=1.3). Abbreviations are phosphatidylethanolamines (PE), lysophosphatidylethanolamines (LPE), phosphatidylcholines (PC), lysophosphatidylcholines (LPC), N-Acylethanolamine (NAE), acylcarnitines (CAR), diacylglycerols (DG), phosphatidylinositol (PI), sphingomyelin (SM), ceramides (Cer), Hexosylceramides (HexCer), phosphatidylserines (PS), Monoradylglycerolipids (MG) and Triradylglycerolipids (TG).

Unknown measured Metabolites

Monisotopic Mass (m/z)	Metabolite name	DRG-conditioned external solution			DRG-conditioned ischemic solution		
		1	2	3	1	2	3
84.9599	Unknown-1	1380	15289	1380	1380	17114	15580
110.0086	Unknown-2	967	22796	967	967	967	9675
119.0858	Unknown-3	6370	6370	6370	6370	6370	68744
124.1120	Unknown-4	1582	16592	19164	18535	1582	15822
125.0956	Unknown-5	2161	25263	2161	2161	25766	21631
145.1219	Unknown-6	5422	222393	196199	5422	138953	220170
150.9768	Unknown-7	2787	2787	2787	2787	2787	2787
152.1067	Unknown-8	3002	34139	3002	36358	36034	31428
160.0423	Unknown-9	1745	1745	1745	1745	1745	1745
160.0423	Unknown-10	19813	19813	19813	19813	19813	19813
166.0869	Unknown-11	34439	30128	40719	2057	20569	25523
170.1169	Unknown-12	125606	1607	1607	1607	1607	1607
171.1499	Unknown-13	1621	16213	1621	17666	1621	1621
176.0701	Unknown-14	1239	1239	1239	1239	1239	1239
188.1289	Unknown-15	2195	27251	2195	2195	32124	24509
196.8656	Unknown-16	38276	42208	30687	2762	2762	2762
198.9398	Unknown-17	1208	26224	22679	1208	13254	18691
203.0523	Unknown-18	1858574	6724	1668140	6724	272890	298850
210.1098	Unknown-19	8516	115817	124676	164732	8516	86210
212.1645	Unknown-20	21746	1545	1545	1545	1545	20330
214.9174	Unknown-21	2084	20835	2084	33457	26709	39991
224.1253	Unknown-22	2843	2843	2843	53817	48692	33074
226.9513	Unknown-23	769	769	769	10365	9231	769

228.2319	Unknown-24	2583	45710	2583	2583	2583	25834
239.1056	Unknown-25	57465	2518328	57465	598450	621606	574653
245.2266	Unknown-26	92172	99296	4757	82755	90033	4757
252.2316	Unknown-29	3794	3794	62508	3794	3794	37943
254.8259	Unknown-30	29719	33420	23867	1883	23074	1883
255.1587	Unknown-31	40604	2057	2057	2057	2057	2057
256.2629	Unknown-32	825768	1939	700308	1939	1939	1939
256.2632	Unknown-33	1377	915901	1377	752067	636591	707817
261.0873	Unknown-35	122132	122132	3580578	1221317	1223689	1320554
263.2365	Unknown-36	223911	13404	13404	13404	13404	13404
265.2528	Unknown-37	287877	13766	268332	13766	13766	13766
265.2529	Unknown-38	18783	283088	18783	256137	246575	265559
266.1724	Unknown-39	1536	35130	1536	1536	28760	16729
268.2269	Unknown-40	3401	3401	3401	3401	3401	3401
269.1384	Unknown-41	52717	2425	72023	2425	44618	41368
280.2629	Unknown-42	5903	5903	5903	5903	59030	5903
280.2639	Unknown-43	869030	1026160	873573	57097	57097	57097
281.1379	Unknown-44	3274	3274	3274	75160	3274	36430
284.2941	Unknown-45	2638	2638	2638	2638	2638	2638
284.9612	Unknown-46	4414	62819	4414	56955	4414	4414
288.9222	Unknown-47	868	868	868	21414	17926	19684
288.9554	Unknown-48	1864	21127	21117	1864	25245	20602
293.2859	Unknown-49	1569	21365	1569	27404	27011	26027
296.2577	Unknown-50	12961	12961	164396	176281	175261	12961
296.2582	Unknown-51	6078	97145	77552	6078	6078	60777
298.2737	Unknown-52	2955	66537	77509	2955	39501	2955
299.0449	Unknown-53	19300	19300	19300	19300	19300	19300

300.2157	Unknown-55	53076	2940	29396	2940	2940	2940
301.0418	Unknown-56	2596	2596	2596	59587	2596	60113
309.1266	Unknown-57	38869	38869	38869	38869	38869	38869
310.3099	Unknown-58	25773	444316	25773	25773	344913	567215
310.3106	Unknown-59	426921	17739	583451	17739	17739	17739
310.8763	Unknown-60	2439	2439	2439	2439	56054	57048
312.3259	Unknown-61	1497	25994	1497	27295	34053	1497
316.2114	Unknown-62	38489	64674	3849	3849	88023	87304
316.2116	Unknown-63	56491	66777	4457	4457	61947	4457
316.2119	Unknown-64	3879	3879	3879	91253	3879	3879
328.8989	Unknown-65	857	14897	12585	11456	9991	12211
334.2348	Unknown-66	8459	8459	8459	8459	8459	8459
335.1055	Unknown-67	1373	1373	1373	1373	1373	1373
338.3408	Unknown-68	179819	9908	147971	9908	9908	137965
338.3413	Unknown-69	1034	1034	1034	1034	1034	1034
338.3429	Unknown-70	11188	128219	11188	130214	117973	11188
341.0289	Unknown-71	15366	316746	288522	153659	172357	175526
343.0265	Unknown-72	112373	120473	106511	6260	64783	65719
344.8754	Unknown-73	845	8825	845	845	19916	20412
350.8927	Unknown-74	901	901	901	10328	9492	9009
351.0578	Unknown-75	835	8407	835	11034	12069	10336
352.1325	Unknown-76	6434	6434	6434	6434	6434	6434
355.063	Unknown-77	1614	1614	1614	1614	1614	1614
357.2145	Unknown-78	1685	22566	21158	1685	21441	1685
357.2383	Unknown-79	1405	14051	1405	29142	27462	22606
361.2222	Unknown-80	18117	1388	15465	1388	1388	1388
366.3728	Unknown-81	2571	50331	2571	41442	2571	2571

367.1311	Unknown-82	6675	6675	6675	6675	6675	6675
367.1315	Unknown-83	2884	2884	2884	2884	2884	2884
381.3006	Unknown-84	23348	1216	24070	1216	1216	1216
389.1147	Unknown-85	7216	7216	7216	7216	7216	7216
397.2016	Unknown-86	48282	2500	46950	2500	2500	41122
398.9876	Unknown-87	264169	253322	232055	126450	12645	12645
400.9848	Unknown-88	8860	8860	157733	89649	90271	91389
402.2426	Unknown-89	2472	2472	2472	2472	2472	2472
412.3207	Unknown-90	2776	126784	2776	2776	2776	2776
412.8642	Unknown-91	873	873	873	8730	10226	9146
415.2109	Unknown-92	1060321	4692	1936745	4692	4692	1255622
416.2277	Unknown-93	6487	78223	6487	76573	6487	77800
419.234	Unknown-94	19283	21685	16015	15504	1521	1521
425.1371	Unknown-95	2075	2075	2075	2075	2075	2075
425.1994	Unknown-96	997	997	997	997	997	997
425.2146	Unknown-97	54959	1881	49772	1881	1881	1881
426.3583	Unknown-98	1669	16692	1669	39955	26390	46352
427.3782	Unknown-99	7520	163831	273708	75198	280084	7520
427.3784	Unknown-100	5650	5650	5650	5650	5650	5650
428.3729	Unknown-101	1688	21423	34101	43898	32299	56366
430.2422	Unknown-102	11005	368008	122319	11005	11005	110054
430.2427	Unknown-103	11263	11263	11263	112630	214672	11263
432.2384	Unknown-104	19561	19561	19561	19561	19561	19561
437.1943	Unknown-105	108166	9752	187452	9752	9752	97938
439.3035	Unknown-106	1341	44987	1341	28537	1341	28148
440.1488	Unknown-107	1680	1680	1680	1680	1680	1680
440.4096	Unknown-108	3551	293499	3551	3551	3551	3551

440.4096	Unknown-109	2641	2641	2641	2641	2641	43605
440.419	Unknown-110	4350	4350	4350	43503	115065	4350
441.3207	Unknown-111	1008	37739	1008	12904	26282	1008
441.7871	Unknown-112	2354	34257	2354	23545	41156	23676
443.3352	Unknown-113	1870	21380	1870	1870	20643	1870
446.2953	Unknown-114	1810	97119	1810	1810	46762	70617
453.0428	Unknown-115	5832	5832	5832	75758	91431	87121
453.1677	Unknown-116	11453	206296	11453	11453	11453	11453
453.1681	Unknown-117	10864	10864	202054	10864	10864	219499
453.3423	Unknown-118	1952	611693	1952	376426	1952	567204
453.3427	Unknown-119	298934	2339	2339	2339	2339	2339
457.2871	Unknown-120	1081	1081	19193	1081	34476	1081
458.8556	Unknown-121	922	9218	922	11383	12897	13751
460.2691	Unknown-122	16414	164144	16414	16414	16414	16414
460.9392	Unknown-123	59598	63514	57540	11890	1189	1189
460.9429	Unknown-124	3225	3225	3225	3225	37580	32854
468.4406	Unknown-125	2875	188963	2875	28754	2875	2875
468.4408	Unknown-126	2521	2521	44938	2521	73234	30876
473.3174	Unknown-127	1554	1554	1554	1554	1554	1554
473.3182	Unknown-128	25993	1206	21116	1206	1206	1206
473.3183	Unknown-129	1199	91353	1199	11988	44643	1199
474.2335	Unknown-130	2064	31141	2064	23924	28267	2064
474.8346	Unknown-131	1057	1057	1057	11482	11368	14037
475.2971	Unknown-132	152375	180822	140752	138070	150178	92244
475.3251	Unknown-133	3375	120270	3375	82752	92878	109836
480.3456	Unknown-134	11728	11728	11728	11728	11728	11728
481.2114	Unknown-135	5496	5496	5496	5496	5496	5496

482.3608	Unknown-136	6639	6639	6639	6639	6639	6639
488.3041	Unknown-137	40140	1786	21923	1786	23466	17865
489.3125	Unknown-138	39790	1787	44939	1787	1787	1787
489.3128	Unknown-139	96416	2638	2638	2638	2638	2638
489.3129	Unknown-140	1166	164343	1166	31027	69411	16894
489.3139	Unknown-141	4159	111615	64934	62918	94238	53540
492.3243	Unknown-142	69875	82899	63684	61100	67663	41768
492.4636	Unknown-143	1571	1571	17054	1571	15714	18669
497.2792	Unknown-144	19019	1313	16796	1313	1313	1313
498.8023	Unknown-145	1321	22843	30506	16067	32454	1321
499.3331	Unknown-146	2102	142866	2102	31204	57445	21020
506.3605	Unknown-147	3237	3237	3237	3237	3237	3237
508.3758	Unknown-148	5688	5688	5688	5688	5688	5688
508.3769	Unknown-149	2467	2467	2467	2467	2467	2467
511.5192	Unknown-150	47327	63928	37020	1012	1012	44101
511.5197	Unknown-151	1644	1644	1644	44520	41139	1644
513.3488	Unknown-152	15850	66224	15020	1289	21384	12886
514.9043	Unknown-153	5534	106893	5534	5534	55336	5534
515.3279	Unknown-154	1119	83826	1119	14613	31238	11192
516.9025	Unknown-155	134502	156816	110469	8012	8012	8012
518.3221	Unknown-156	2222	2222	2222	2222	2222	22225
518.8987	Unknown-157	3471	71299	3471	38412	37070	36566
520.3546	Unknown-158	24842	36787	34713	37787	34491	21935
520.5076	Unknown-159	3291	417760	3291	3291	3291	3291
526.4315	Unknown-160	3708	116351	3708	135557	154103	73606
528.2445	Unknown-161	11698	220044	11698	221908	237096	233232
528.2446	Unknown-162	225830	7628	241910	7628	7628	7628

534.3714	Unknown-163	1210	18315	12098	1210	1210	1210
534.3715	Unknown-164	13506	1351	1351	1351	1351	1351
536.1649	Unknown-165	3428	3428	46485	3428	3428	3428
536.1652	Unknown-166	3659	3659	3659	3659	38062	3659
536.4885	Unknown-167	1942	1942	21235	1942	1942	1942
537.1399	Unknown-168	57692	64202	58303	5769	5769	5769
538.3858	Unknown-169	4158	4158	4158	4158	4158	4158
540.4241	Unknown-170	699603	1320552	339941	27119	27119	306889
540.4254	Unknown-171	11401	11401	11401	11401	11401	11401
542.2603	Unknown-172	9875	228486	239487	224313	237584	238776
544.3393	Unknown-173	2736	2736	2736	27697	2736	27362
550.3867	Unknown-174	2031	2031	2031	2031	2031	20313
554.4626	Unknown-175	1681	39114	1681	52018	63401	34235
556.2756	Unknown-176	153705	3930	3930	3930	3930	3930
559.1246	Unknown-177	4718	4718	4718	81469	4718	4718
559.1253	Unknown-178	21395	2129	2129	2129	89000	92325
559.5195	Unknown-179	4162	125113	115607	102929	4162	4162
563.5507	Unknown-180	678870	651192	620250	27167	27167	534557
567.5814	Unknown-181	1803	51140	43314	48366	43981	41574
568.4565	Unknown-182	19289	902560	225934	19289	619431	19289
568.4571	Unknown-183	426740	14245	14245	14245	14245	236789
568.4576	Unknown-184	6186	6186	6186	6186	6186	6186
568.4782	Unknown-185	35916	26854	56098	19746	41692	37758
570.2922	Unknown-186	2835	73949	69239	2835	59559	67861
570.4581	Unknown-187	6157	198286	6157	241291	230471	146722
574.2854	Unknown-189	1247	1247	25043	1247	1247	1247
574.2869	Unknown-190	1400	37840	1400	22079	29634	14005

574.8599	Unknown-191	5694	5694	94830	58874	57310	5694
574.8604	Unknown-192	101762	105993	5205	5205	5205	56002
575.1296	Unknown-193	2526	2526	2526	2526	2526	2526
576.8588	Unknown-194	67848	71620	3028	34656	3028	3028
599.3901	Unknown-195	3860	59996	3860	38605	88434	38872
599.3903	Unknown-196	4622	4622	4622	49931	58944	46222
608.3842	Unknown-198	1661	43449	48739	1661	1661	41612
610.1829	Unknown-199	8341	8341	8341	8341	1284291	8341
610.1842	Unknown-200	4690	4690	93626	4690	4690	46900
611.1387	Unknown-201	852	852	852	17084	16241	8524
612.5046	Unknown-202	1322	29650	1322	25674	48352	38918
614.4827	Unknown-203	9766	278240	9766	402222	424278	9766
619.6141	Unknown-204	2110	61201	2110	61456	64042	56088
627.5342	Unknown-205	2631	2631	2631	2631	2631	240176
627.5345	Unknown-206	2418	2418	127922	2418	236443	2418
630.8218	Unknown-207	35951	34938	23660	2094	2094	2094
632.8193	Unknown-208	63852	69268	3486	34857	3486	36448
640.3923	Unknown-209	1449	1449	20345	1449	14779	1449
655.1658	Unknown-210	7457	7457	7457	74569	89753	90494
656.5297	Unknown-211	33156	1270	56170	1270	1270	42552
658.5099	Unknown-212	381621	334356	556718	12170	12170	404840
661.0637	Unknown-213	1871	63202	64846	1871	21871	1871
663.4528	Unknown-214	35488	35488	35488	35488	35488	35488
670.5469	Unknown-215	1238	36235	1238	30995	54917	37521
687.2152	Unknown-216	4760	4760	4760	4760	4760	4760
690.7797	Unknown-217	2392	51084	2392	2392	2392	29052
699.4072	Unknown-218	2459	29366	2459	24587	37319	29967

702.5362	Unknown-219	9601	308134	9601	340460	391272	388825
708.5125	Unknown-220	7591	7591	7591	7591	7591	7591
713.4206	Unknown-221	3445	99385	3445	61778	117615	34454
713.4219	Unknown-222	6831	6831	104149	6831	6831	6831
746.5615	Unknown-223	9721	295707	443988	9721	349711	322951
774.5918	Unknown-224	3931	102608	213125	144762	180792	158811
775.2346	Unknown-225	34736	34425	34960	3443	3443	3443
790.5862	Unknown-226	7330	211818	331183	201622	294393	261896
803.2322	Unknown-227	967	11560	967	22121	23381	11519
818.6186	Unknown-228	84333	68242	155349	92726	131485	104655
834.6138	Unknown-229	4634	121657	4634	143934	179958	183110
850.2502	Unknown-230	144544	40994	2788	89208	2788	2788
862.6446	Unknown-231	1589	40568	1589	56511	75988	67556
915.2489	Unknown-232	2450	2450	2450	24504	33260	31178
922.6669	Unknown-233	1105	35858	62629	28832	37849	50631
996.6011	Unknown-234	1317	13173	1317	13548	26137	1317

Supplementary Table S2: Unknown measured metabolites. A total of 234 metabolites remain unknown after being measured in positive mode with values for each measured sample shown. Among these, 15 were significantly regulated. The list is sorted by monisotopic mass (m/z).

References

1 Zhou, G. *et al.* NetworkAnalyst 3.0: a visual analytics platform for comprehensive gene expression profiling and meta-analysis. *Nucleic Acids Res* **47**, W234-w241 (2019). https://doi.org/10.1093/nar/gkz240
